# Food availability as a major driver in the evolution of life‐history strategies of sibling species

**DOI:** 10.1002/ece3.2909

**Published:** 2017-04-28

**Authors:** Raphaël Arlettaz, Philippe Christe, Michael Schaub

**Affiliations:** ^1^ Institute of Ecology and Evolution – Division of Conservation BiologyUniversity of BernBernSwitzerland; ^2^ Swiss Ornithological InstituteSempachSwitzerland; ^3^ Grupo de Investigaciones de la BiodiversidadIADIZACONICET–CCTMendozaArgentina; ^4^ Department of Ecology and EvolutionUniversity of Lausanne, BiophoreLausanneSwitzerland

**Keywords:** age at first reproduction, bats, demography, life‐history trade‐off, multistate capture–recapture model, survival

## Abstract

Life‐history theory predicts trade‐offs between reproductive and survival traits such that different strategies or environmental constraints may yield comparable lifetime reproductive success among conspecifics. Food availability is one of the most important environmental factors shaping developmental processes. It notably affects key life‐history components such as reproduction and survival prospect. We investigated whether food resource availability could also operate as an ultimate driver of life‐history strategy variation between species. During 13 years, we marked and recaptured young and adult sibling mouse‐eared bats (*Myotis myotis* and *Myotis blythii*) at sympatric colonial sites. We tested whether distinct, species‐specific trophic niches and food availability patterns may drive interspecific differences in key life‐history components such as age at first reproduction and survival. We took advantage of a quasi‐experimental setting in which prey availability for the two species varies between years (pulse vs. nonpulse resource years), modeling mark‐recapture data for demographic comparisons. Prey availability dictated both adult survival and age at first reproduction. The bat species facing a more abundant and predictable food supply early in the season started its reproductive life earlier and showed a lower adult survival probability than the species subjected to more limited and less predictable food supply, while lifetime reproductive success was comparable in both species. The observed life‐history trade‐off indicates that temporal patterns in food availability can drive evolutionary divergence in life‐history strategies among sympatric sibling species.

## 1 Introduction

Life‐history theory provides a framework for understanding how evolution shapes life cycles, where natural selection is the ultimate driver of species‐specific vital rates and breeding tactics (Stearns, 1976). Central to this theory is the resource allocation trade‐off between an individual's own maintenance, which affects growth and survival, and the effort exerted to maximize reproductive output (Stearns, 1992). In long‐lived iteroparous species, age at first reproduction appears to be nodal. In effect, natural selection for survival is strong before individuals of these species start reproducing. Thereafter, natural selection favors high and mostly constant survival rates within the reproducing segment of the population (Eberhardt, 2002; Gaillard & Yoccoz, 2003; Jones et al., 2008). Finally, beyond the active reproductive phase, when symptoms of aging begin to manifest, decreased survival probabilities are observed (Bize, Devevey, Monaghan, Doligez, & Christe, 2008; Bize et al., 2014; Gaillard, Festa‐Bianchet, Yoccoz, Loison, & Toigo, 2000). An early reproductive start thus has the advantage of reducing the risk of dying before reproduction, however, at the risk of lower offspring quality and/or reduced litter size. In contrast, a late reproductive start increases the risk of dying during the longer immature phase, but can yield larger litter sizes and offspring of superior quality (Stearns, 1992). Weighting up the different life‐history strategies, each may result in fairly similar fitness such that life‐history variations are eventually maintained high by natural selection (Schmidt, Hoedl, & Schaub, 2012).

Energy input remains the crux for individual decisions in resource allocation trade‐offs among different life‐history components. As variation in food availability is the main determinant of energy supply for any given organism, it might be considered an ultimate driver of life‐history evolution (Lack, 1954). This is evidenced by the widespread observation that when food availability is temporally low, first reproduction is delayed, and subsequently, survival and reproductive output are reduced (Brommer, Pietiainen, & Kolunen, 1998; Karell, Ahola, Karstinen, Zolei, & Brommer, 2009). Hence, individuals that have more limited access to food supply (lower food quantity overall, more pronounced temporary bottlenecks in food availability, etc.) are expected to have a more conservative, slower life‐history strategy, while the opposite is true when food supply is high (Roff, 1980). It remains to be demonstrated whether patterns of food resource availability and exploitation can also explain interspecific differences in life history acquired in parallel to niche differentiation. Controlled experiments where food is alternately added or suppressed are required to evaluate reactiveness of life‐history tactics to such changes. However, conducting fully controlled experiments over relevant evolutionary time frames is mostly unrealistic with long‐lived species. Quasi‐experiments make use of specific natural contexts where food availability varies within time and/or space, thereby providing a way to overcome this methodological limitation. Here, we used a quasi‐experimental situation to assess how life‐history strategies and demography of two sibling bat species might be modulated by differential food availability.

Sibling species are phylogenetically more related to each other than to any other species. These species share the same evolutionary history, having diverged from a recent common ancestor. The advantage of working with sibling species, at least in areas where they have occurred in sympatry for generations, is that observed interspecific differences are most likely the result of diverging evolutionary trajectories. Their species‐specific morphological, ecological, or behavioral traits can therefore be seen as recent (post‐speciation) adaptations to different niche contexts, unless they were the root cause of speciation by disruptive selection. Whatever the mechanism of speciation, this allows strong inferences to be made about how natural selection drives the evolution of life‐history traits between species.

Long‐term, individual‐based studies are commonly used to investigate the consequences of various environmental factors on life‐history traits (Clutton‐Brock & Sheldon, 2010). We individually ringed juveniles and adults of two recently speciated sympatric sibling species of insectivorous bats and monitored their reproductive parameters and dispersal (Arlettaz, Ruedi, Ibanez, Palmeirim, & Hausser, 1997; Ruedi & Mayer, 2001) with the objective to evaluate whether subtle species‐specific ecological differences translate into distinct life‐history adjustments. These two bat species share common nursery roosts in their wide area of sympatry (Arlettaz, Ruedi, et al., 1997), where they coexist in a stable way. This is possible thanks to a clear‐cut niche resource partitioning mechanism along the foraging habitat and diet axes (Arlettaz, 1999; Arlettaz, Perrin, & Hausser, 1997) although the two species behave as generalist predators (Arlettaz & Perrin, 1995). One species, the greater mouse‐eared bat *Myotis myotis* (Borkhausen 1797) exploits primarily ground‐dwelling invertebrates, mostly carabid beetles (Coleoptera, Carabidae) that occur as fully grown adults at a fairly constant rate from early spring until the onset of hibernation (Arlettaz, Christe, Lugon, Perrin, & Vogel, 2001; Arlettaz & Perrin, 1995). Its food supply appears thus fairly predictable. In contrast, its sister taxon, the lesser mouse‐eared bat *Myotis blythii* (Tomes 1857) relies on bush crickets (Saltatoria, Tettigoniidae). Bush crickets are a profitable prey when they reach adult body size, that is, from late spring or early summer onward, but are not in early spring (Arlettaz & Perrin, 1995; Arlettaz, Perrin, et al., 1997; Arlettaz et al., 2001). The two bat species thus usually face marked seasonal differences in food supply in spite of their great similarity in morphology, foraging, and roosting behavior (Arlettaz, 1999; Arlettaz, Perrin, et al., 1997). The delayed availability of the main food source of the lesser mouse‐eared bat (bush crickets), that is, the lower food supply it usually experiences in early spring, results in a median delay in parturition of approximately 10 days compared to the greater mouse‐eared bat (Arlettaz et al., 2001). Such a temporal difference may have an impact on the age at first reproduction because mating in both species takes place in late summer, with late‐born pups being unlikely to reach sexual maturity as yearlings (Frick, Reynolds, & Kunz, 2010). Thus, the lesser mouse‐eared bat is more likely to have delayed sexual maturation compared to the greater mouse‐eared bat (Arlettaz, Baeriswyl, Christe, & Lugon, 1998)**.** Yet, the interspecific difference in parturition times disappears in years with massive occurrence of cockchafers *Melolontha melolontha* Fabricius 1775 (Arlettaz et al., 2001), which takes place every third or fourth year from late April to early June in the study area. In such years, cockchafers are a superabundant, though temporary food resource for both species (Arlettaz & Perrin, 1995; Arlettaz, Perrin, et al., 1997). Cockchafers thus represent a typical pulse resource, as described for various ecosystems and taxa. This pulse resource, which is less exploited by the greater than by the lesser mouse‐eared bat (Arlettaz, Ruedi, et al., 1997), offers an opportunity for lesser mouse‐eared bats to compensate for the lack of their favorite food, bush crickets, in early spring, and therefore to advance parturition compared to non‐cockchafer years: Births become then synchronous in the two bat species in cockchafer years.

Based on this knowledge, we made the following predictions. First, we assumed a later age at first reproduction in lesser mouse‐eared bats compared to greater mouse‐eared bats. In effect, lesser mouse‐eared bats in non‐cockchafer years experience less abundant food supply than in cockchafer years, which also means that their prey is also less predictable across the years. This reduced food availability in non‐cockchafer years systematically delays parturition in the lesser mouse‐eared bat, which is also likely to compromise achieving sexual maturity during their first year of life (Arlettaz et al., 1998, 2001). Second, assuming our first prediction would be correct, we predicted higher adult survival probabilities for the lesser mouse‐eared bat compared to the greater mouse‐eared bat due to classical life‐history trade‐offs between survival and reproduction (Promislow & Harvey, 1990). In contrast, we did not expect to find interspecific differences in first‐year survival probabilities. In effect, although life‐history theory predicts lower juvenile mortality with increasing age at first reproduction (Stearns, 1992), these effects are generally small (Stearns, 1992). Life‐history theory also predicts reduced temporal variations in life‐history traits that have a high impact on fitness (high demographic sensitivity) compared to variations in less fitness‐relevant traits (Pfister, 1998). As first‐year survival has a lower fitness sensitivity than adult survival in long‐lived species (Millar & Zammuto, 1983; Oli & Dobson, 2003; Saether & Bakke, 2000)**,** and mouse‐eared bats are typically long‐lived creatures (Arlettaz, Christe, & Desfayes, 2002; Wilkinson & South, 2002), we expected that first‐year survival in both species would vary more over time than adult survival (Schorcht, Bontadina, & Schaub, 2009). Our quasi‐experimental situation, with years with and without cockchafers, also provided room for testing a third prediction, namely that the two species would be demographically more similar in cockchafer years. Cohorts of the two species should no longer differ in survival and age at first reproduction if born during peak cockchafer years. In addition, and this is our fourth prediction, we expected higher survival probabilities in both species in cockchafer years because this superabundant food source is likely to boost both bat species (Arlettaz, Perrin, et al., 1997). Finally, we predicted dispersal movements of reproducing adults between the two study nursery roosts according to the local availability of cockchafers.

This study in essence investigated whether food resource availability could operate as an ultimate driver of life‐history strategies. To achieve state‐of‐the‐art analysis, we relied on probabilistic capture–recapture models that account for imperfect detection and deliver accurate estimates of life‐history traits.

## 2 Material and Methods

### 2.1 Data sampling

Bats were mist‐netted at least four times during the active season (May‐August) from 1989 to 2001 (except in 1994) at two colonial roosts (Raron and Naters; 46°18′N, 7°48′‐7°59′E; distance between roosts: 14.5 km) in the upper Rhône valley (Valais, Switzerland). Every captured female was inspected for age. We distinguished two age classes: young (born in the current calendar year: cartilaginous metacarpial joints; born the year before: presence of a gray chin spot) and adults (individuals showing no cartilaginous joints and no gray chin spot). Note that the age of the individuals marked as adults cannot be exactly known. We also recorded the reproductive status of all captured females as reproducing (embryo detected in abdomen cavity via palpation or milk extractable from mammary glands) and nonreproducing (no embryo detected in abdomen cavity or no milk extractable from mammary glands). Each individual was marked with a forearm ring.

Mass occurrence of cockchafers was assessed yearly in May by visiting mouse‐eared bats’ traditional foraging grounds located by radiotracking (Arlettaz, 1999). Cockchafers show a patchy spatial distribution in Valais, reaching high densities in grassland interspersed with hedges and isolated deciduous trees. Around Raron, cockchafers show a typical 3‐year cycle (observed in 1989, 1992, 1995, 1998 and 2001), whereas in the vicinity of Naters, massive flights occur every fourth year only (observed in 1990, 1994 and 1998; in 1998 the two areas experienced mass occurrences simultaneously). This difference is likely due to slightly diverging local environmental conditions, with warmer temperatures and lower precipitation levels in Raron. Overall, cockchafers in the study area were present every second year.

Bats of the two colonies sometimes exchange individuals, although they do not do so with the next colony that is located 60 km to the East. This finding is based on observations made on the 3,953 mouse‐eared bats ring‐tagged at the three known Valais nursery roosts since 1948 (R. Arlettaz, unpublished data).

### 2.2 Statistical analyses

#### 2.2.1 Multistate capture–recapture model

We used a multistate capture–recapture model to estimate the probabilities of apparent survival (ϕ), first reproduction (α), natal dispersal (*n*), breeding dispersal (*b*), and recapture (*p*) from the capture–recapture data. This model is similar to the model introduced by Lebreton, Hines, Pradel, Nichols, and Spendelow (2003). We defined four states (1: young individuals present in Naters; 2: young individuals present in Raron; 3: experienced breeders present in Naters; 4: experienced breeders present in Raron) and modeled transition among states with the target parameters. Individuals born at the studied nursery colonies (i.e., that were marked as juvenile individuals) were assigned to states 1 or 2, depending on location. At and after their first observed reproduction, they were assigned to states 3 or 4, again depending on location. Individuals that were marked as adults were always assigned to states 3 or 4, regardless of whether they were observed reproducing in the current year. The data are summarized in individual capture histories, where 0 indicates an individual that has not been captured in the corresponding year, and 1–4 an individual that has been captured and was in state 1–4 as defined above. These data were analyzed with a multistate capture–recapture model parameterized in terms of 4 × 4 transition matrices (states at time *t* are in rows, states at time *t *+* *1 in columns) and of 4 × 1 vectors of recapture probabilities. As some model parameters change with age and some transitions are restricted to specific age classes, we used age‐specific transition matrices. The model requires that the age at which all bats have started to reproduce is fixed; otherwise some parameters are not estimable (Lebreton et al., 2003). We fixed this age to 4 years, because we never observed an individual starting to reproduce later than at 4 years. The transition matrix and the recapture vector from age 0 to age 1 year are as follows:

$$\left[\begin{array}{cccc} \phi_{\mathrm{juv}}^{N}\left(1-n^{\mathrm{NR}}\right)\left(1-\alpha_{1}^{N}\right) & \phi_{\mathrm{juv}}^{N}n^{\mathrm{NR}}\left(1-\alpha_{1}^{R}\right) & \phi_{\mathrm{juv}}^{N}\left(1-n^{\mathrm{NR}}\right)\alpha_{1}^{N} & \phi_{\mathrm{juv}}^{N}n^{\mathrm{NR}}\alpha_{1}^{R}\\ \phi_{\mathrm{juv}}^{R}n^{\mathrm{RN}}\left(1-\alpha_{1}^{N}\right) & \phi_{\mathrm{juv}}^{R}\left(1-n^{\mathrm{RN}}\right)\left(1-\alpha_{1}^{R}\right) & \phi_{\mathrm{juv}}^{R}n^{\mathrm{RN}}\alpha_{1}^{N} & \phi_{\mathrm{juv}}^{R}\left(1-n^{\mathrm{RN}}\right)\alpha_{1}^{R}\\ 0 & 0 & 0 & 0\\ 0 & 0 & 0 & 0 \end{array}\right]\left[\begin{array}{c} p_{\mathrm{nb}}^{N}\\ p_{\mathrm{nb}}^{R}\\ p_{\mathrm{br}}^{N}\\ p_{\mathrm{br}}^{R} \end{array}\right],$$

the transition matrix and the recapture vector from age *j* to *j *+* *1 (1 ≤ *j *≤* *4):

$$\left[\begin{array}{cccc} \phi_{\mathrm{ad}}^{N}\left(1-b^{\mathrm{NR}}\right)\left(1-\alpha_{j}^{N}\right) & \phi_{\mathrm{ad}}^{N}b^{\mathrm{NR}}\left(1-\alpha_{j}^{R}\right) & \phi_{\mathrm{ad}}^{N}\left(1-b^{\mathrm{NR}}\right)\alpha_{j}^{N} & \phi_{\mathrm{ad}}^{N}b^{\mathrm{NR}}\alpha_{j}^{R}\\ \phi_{\mathrm{ad}}^{R}b^{\mathrm{RN}}\left(1-\alpha_{j}^{N}\right) & \phi_{\mathrm{ad}}^{R}\left(1-b^{\mathrm{RN}}\right)\left(1-\alpha_{j}^{R}\right) & \phi_{\mathrm{ad}}^{R}b^{\mathrm{RN}}\alpha_{j}^{N} & \phi_{\mathrm{ad}}^{R}\left(1-b^{\mathrm{RN}}\right)\alpha_{j}^{R}\\ 0 & 0 & \phi_{\mathrm{ad}}^{N}\left(1-b^{\mathrm{NR}}\right) & \phi_{\mathrm{ad}}^{N}b^{\mathrm{NR}}\\ 0 & 0 & \phi_{\mathrm{ad}}^{R}b^{\mathrm{RN}} & \phi_{\mathrm{ad}}^{R}\left(1-b^{\mathrm{RN}}\right) \end{array}\right]\left[\begin{array}{c} p_{\mathrm{nb}}^{N}\\ p_{\mathrm{nb}}^{R}\\ p_{\mathrm{br}}^{N}\\ p_{br}^{R} \end{array}\right],$$

the transition matrix and the recapture vector from age 4 to age 5 years:

$$\left[\begin{array}{cccc} 0 & 0 & \phi_{\mathrm{ad}}^{N}\left(1-b^{\mathrm{NR}}\right) & \phi_{\mathrm{ad}}^{N}b^{\mathrm{NR}}\\ 0 & 0 & \phi_{\mathrm{ad}}^{R}b^{\mathrm{RN}} & \phi_{\mathrm{ad}}^{R}\left(1-b^{\mathrm{RN}}\right)\\ 0 & 0 & \phi_{\mathrm{ad}}^{N}\left(1-b^{\mathrm{NR}}\right) & \phi_{\mathrm{ad}}^{N}b^{\mathrm{NR}}\\ 0 & 0 & \phi_{\mathrm{ad}}^{R}b^{\mathrm{RN}} & \phi_{\mathrm{ad}}^{R}\left(1-b^{\mathrm{RN}}\right) \end{array}\right]\left[\begin{array}{c} 0\\ 0\\ p_{\mathrm{br}}^{N}\\ p_{\mathrm{br}}^{R} \end{array}\right],$$

and the transition matrix and the recapture vector from age 5 years onward, which is also relevant for individuals marked as adults:

$$\left[\begin{array}{cccc} 0 & 0 & 0 & 0\\ 0 & 0 & 0 & 0\\ 0 & 0 & \phi_{\mathrm{ad}}^{N}\left(1-b^{\mathrm{NR}}\right) & \phi_{\mathrm{ad}}^{N}b^{\mathrm{NR}}\\ 0 & 0 & \phi_{\mathrm{ad}}^{R}b^{\mathrm{RN}} & \phi_{\mathrm{ad}}^{R}\left(1-b^{\mathrm{RN}}\right) \end{array}\right]\left[\begin{array}{c} 0\\ 0\\ p_{\mathrm{br}}^{N}\\ p_{\mathrm{br}}^{R} \end{array}\right].$$

The superscripts of the model parameters indicate to which colony they are specific (*N*: Naters; *R*: Raron) and the movement between colonies (NR: movement from Naters to Raron; RN: movement from Raron to Naters). The subscripts refer to age (juv: from age 0 to 1 year old; ad: at least 1 year old; 1: exactly 1 year old; *j*: at age *j* years old) and to reproducing status (br: experienced breeder; nb: individual that has not yet reproduced). Note that the individuals marked as adults only provide information about adult survival, breeding dispersal adult recapture probability, while individuals marked as juveniles provide information about all parameters of the model.

The capture histories are mutually exclusive events, and thus, the observed number of individuals with the various capture histories follows a multinomial distribution. Maximum likelihood methods were used to estimate the parameters.

#### 2.2.2 Candidate models for differences between species

The model selection strategy is described in detail in Supplementary online material. We considered five models for the recapture probability. Recapture probabilities were time‐dependent in all models as the capture effort was not the same in all years. Moreover, we did not capture bats in 1994 and the corresponding recapture probability was fixed to zero in all models. We did not test whether the recapture probabilities were the same for both colonies, because in 2001, the Raron colony could only be sampled at low intensity as their roost (an attic) was undergoing renovation. Hence, the interaction colony*time had to be kept in the model. However, we did include models with and without effects of species and reproductive status.

To model natal and breeding dispersal between the two nursery roosts (movement), we considered four models. We formulated models where natal and breeding dispersal were both species‐specific; where only one of the two was species‐specific; and where both were the same in the two species. We did not consider models with year effects because we were not interested in such effects on dispersal and due to the sparseness of available data.

To model the probability to start reproduction, we considered four different models and only young females with known age were informative about this parameter. We assumed that the probability to start to reproduce was constant over time. This was enforced due to the sparseness of the data, but we will relax this assumption in the second modeling step. The models considered differed as to whether or not effects of species and nursery roosts (colonial site) were present. Comparison between these models enabled to evaluate whether the probability to start to reproduce depended on the colony of birth (origin) and species.

Survival probabilities were modeled with 14 different models. We always considered two age classes: the first referred to the first year of life and the second to all ages beyond the first year. The fitted models differed as to whether or not year‐specific variations occurred in both age classes or in the first age class only, whether survival probabilities differed between species, and whether colony effects were present. Moreover, we included models in which the temporal variation was different between species (interacting models) and models in which temporal variation was the same (additive models and models without species‐specific parameters). Comparing these models enabled us to evaluate whether the two species were similarly sensitive to environmental variation.

#### 2.2.3 Candidate models for the effect of cockchafer years

In the following modeling exercise, we evaluated whether survival and age‐specific probability of first reproduction were impacted by cyclic food conditions such as the mass occurrence of cockchafers in some years. The constructed models were based on the most parsimonious structure identified during the preceding modeling. We fitted models in which the target parameters were a function of whether a specific year was linked or not with mass occurrence of cockchafers. We included models where the target parameters of both species were a function of cockchafer years, and where only one species was impacted. This way it was possible to assess whether both species were similarly affected by cyclic food conditions. For the age‐specific probability of first reproduction, we included the effect of cockchafers as a cohort as well as a temporal effect. For the probability of starting to reproduce beyond the first year, this makes a difference: The cohort model tests for long‐lasting effects of cyclic food conditions; that is, it assumes that the probability to start to reproduce is different for individuals born in a cockchafer year vs. a non‐cockchafer year. By contrast, in the temporal model, the year of birth is assumed to only affect the current year. This model therefore simply assumes that the probability to start to reproduce differs depending on whether it occurs in a cockchafer year or not.

For all of the models, we considered common cockchafer years across the whole study area. Initially, we also tested for colony‐specific cockchafer years, but these yielded similar results, which is why they are not presented here. From the parameter estimates, we calculated the mean age at first reproduction as

$$\text{AFR}=\alpha_{1}+\sum_{n=2}^{4}\left(n\alpha_{n}\prod_{i=1}^{n-1}\left(1-\alpha_{i}\right)\right).$$

## 3 Results

During the 13‐year study period, we marked 430 and 849 female lesser and greater mouse‐eared bats, respectively, of which 227 and 461, respectively, were individuals of exact known age. The goodness‐of‐fit test of a general multistate model was acceptable (*χ* ^2^_207_ = 201.12, *p *=* *.60).

### 3.1 Differences between species

Starting from a complex multistate model, we reduced the complexity in a stepwise manner. In the results below, we first present overall model results, then briefly describe main summary statistics for parameters of major interest according to modeling outcomes. The best model for recapture probability included an additive effect of species and reproduction on roost, and year‐specific effects (Appendix S1, Table S1).

Model selection for movement between nursery roosts clearly revealed a species‐specific dispersal probability for adults, while this was less clear for first‐year individuals (Appendix S1, Table S2).

There was no evidence that the age‐specific probability to reproduce for the first time differed between colonies, although strong evidence pointed toward this being species‐specific (Appendix S1, Table S3).

The best model included a species effect that was additive on the age‐specific probability for starting reproduction. Modeling survival showed that adult survival was constant across time but species‐specific. Juvenile survival was highly variable across time, but there was some uncertainty about a species effect (Table 1). The top three models had no species effect, while the ensuing models did reveal an additive species effect.

**Table 1:** Selection among different survival probability models of *Myotis myotis* and *Myotis blythii* at the colonies of Naters and Raron. The model for age‐specific first time reproduction (α_a3+species_) was always the same and therefore not included in the list below. We present the model's deviance, the number of estimated parameters, the difference in the Akaike's information criterion between the actual and the best model (ΔAIC), and the Akaike's weight

Survival model (ϕ)	Movement model (ψ)	Recapture model (*p*)	Deviance	Parameters	ΔAIC	Weight
juv: year; ad: spec	juv:.; ad: spec	Col*year+rep+spec	8749.19	51	0.00	0.212
juv: year; ad: spec	a2*spec	Col*year+rep+spec	8745.79	53	0.60	0.157
juv: year; ad: spec	juv:.; ad: spec	Col*year*rep	8710.05	71	0.86	0.138
juv: year+spec; ad: spec	juv:.; ad: spec	Col*year+rep+spec	8749.12	52	1.93	0.081
juv: year+spec; ad: spec	juv:.; ad: spec	Col*year*rep	8709.25	72	2.06	0.076
juv: year+spec; ad: spec	a2*spec	Col*year+rep+spec	8745.61	54	2.42	0.063
juv: year*spec; ad: spec	juv:.; ad: spec	Col*year*rep	8690.11	82	2.92	0.049
juv: year; ad: .	juv:.; ad: spec	Col*year+rep+spec	8754.83	50	3.64	0.034
juv: year; ad: .	a2*spec	Col*year+rep+spec	8751.29	52	4.10	0.027
a2*spec	a2*spec	Col*year+rep+spec	8767.44	44	4.25	0.025
a2*spec	juv:.; ad: spec	Col*year+rep+spec	8771.52	42	4.33	0.024
juv: year*spec; ad: spec	juv:.; ad: spec	Col*year+rep+spec	8731.57	62	4.39	0.024

Model notation: rep: individuals that have reproduced at least once and those that have not started to reproduce yet differ; a2: 2 age classes (1st year, later); year: different parameter for each year; Col: different parameter for each colony; spec: different parameter for each species; juv: juveniles (1st year); ad: adults (at least 1 year old); * interactive effects; + additive effects; . is for constancy. Shown are the best (weight > 0.02) of 56 fitted models.

**Figure 1:**
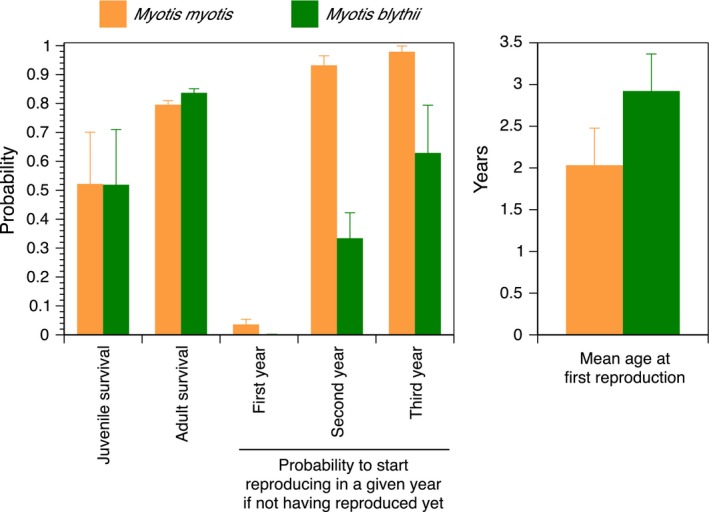
Mean model‐averaged (across all models of Table 1 with Akaike weight > 0.02) demographic rates of *Myotis myotis* and *Myotis blythii*. Given are mean values and unconditional standard errors. Note that for juvenile survival, we provide the geometric mean because this rate was year‐specific in the best models. For reproduction, the probability shown is that of a given female that has not yet reproduced to start reproducing in a given year (1st, 2nd, or 3rd year)

The model‐averaged life‐history traits for both species are presented in Figure 1 and Table 2. In average, lesser mouse‐eared bats started to reproduce at a mean age of 2.92 (± 1.88) years (unconditional standard error of the mean) and had an average annual survival probability of 0.84 (± 0.01), while greater mouse‐eared bats started to reproduce at 2.03 (± 0.79) years of age and achieved an annual adult survival probability of 0.80 (± 0.01). The probability to reproduce for the first time during the first year of life was very low in both species. In the second year, it averaged 0.93 (± 0.03) and 0.33 (± 0.09) in greater and lesser mouse‐eared bats, respectively. During the third year, it was, respectively, 0.98 (± 0.02) and 0.63 (± 0.17). Juvenile survival probabilities for both species varied strongly over time, showing parallel changes over the years in both species as well as a similar species‐specific rate: 0.52 (± 0.18 and 0.19, respectively) (Figure 1). Dispersal probabilities varied between 0.02 and 0.17, depending on species and age class (natal dispersal was slightly lower than breeding dispersal; Table 2). Overall, breeding dispersal probabilities were spatially asymmetric in the two species. Greater mouse‐eared bats predominantly moved from Naters to Raron (0.17; opposite direction: 0.05), while lesser mouse‐eared bats mostly went from Raron to Naters (0.14; opposite direction: 0.06).

**Table 2:** Mean model‐averaged (across all models of Table 1 with Akaike weight > 0.02) probability of movements between the two nursery roosts for juvenile and adult *Myotis blythii* and *Myotis myotis*. Given are mean values and unconditional standard errors

Parameter	Greater mouse‐eared bat (*M. myotis*)	Lesser mouse‐eared bat (*M. blythii*)
Movement probability from Raron to Naters, juveniles	0.098 (0.028)	0.108 (0.039)
Movement probability from Naters to Raron, juveniles	0.060 (0.071)	0.019 (0.026)
Movement probability from Raron to Naters, adults	0.053 (0.009)	0.142 (0.022)
Movement probability from Naters to Raron, adults	0.172 (0.035)	0.063 (0.016)

### 3.2 Impact of cockchafer years

**Figure 2:**
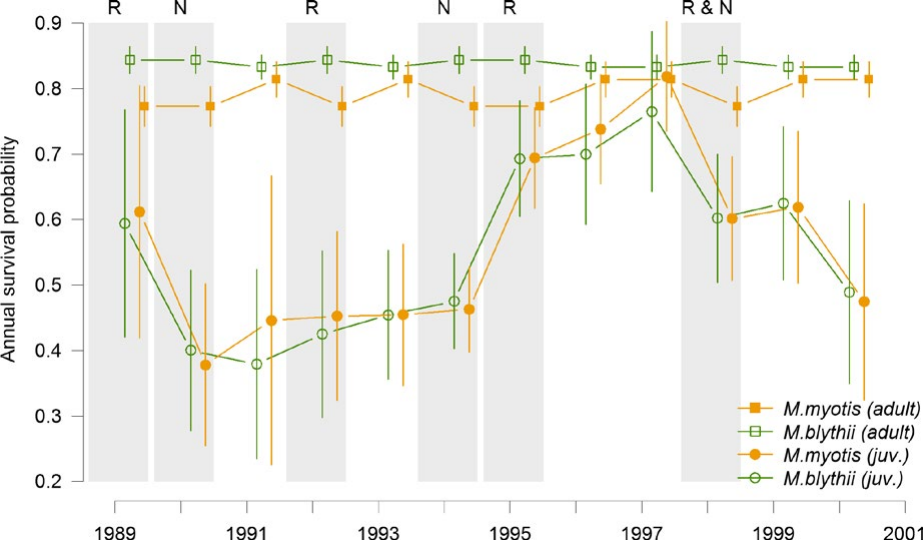
Model‐averaged (across models from Table 3 with Akaike weight > 0.02) annual survival probabilities of lesser (*Myotis blythii*) and greater (*Myotis myotis*) mouse‐eared bats. Open green symbols refer to lesser mouse‐eared bats, closed orange symbols to greater mouse‐eared bats, squares refer to adults (at least 1 year old) and circles refer to first‐year individuals (from weaning until age 1 year). The vertical bars show the limit of the 95% confidence intervals. The shaded areas indicate years with mass occurrence of cockchafers (pulse resource years), with their local spatial occurrence (N: surroundings of Naters nursery roost; R: surroundings of Raron nursery roost; note the occurrence around the two nursery roosts in 1998)

For juvenile survival, mass cockchafer years had no impact on the top models (Table 3). For adult survival, the best model established such an effect for the greater mouse‐eared bats, but did not show any for the second best model for either species. In most (11 of 14 models) of the subsequent best‐ranked models, a mass cockchafer year effect was evident, but with alternated patterns between species: Either both species were affected concurrently (four of 14 models), or singly (four and three models of 14 for lesser and greater mouse‐eared bats, respectively). Model‐averaged estimates of first‐year survival probabilities again suggested that these were not affected by mass cockchafer occurrence. As regards adults, model‐averaged estimates revealed that survival probabilities for adult females of greater mouse‐eared bats were higher during mass cockchafer years (Figure 2). By contrast, adult survival probabilities for female lesser mouse‐eared bats appeared to be slightly lower during mass cockchafer years.

**Table 3:** Modeling first‐year and adult survival probabilities for *Myotis myotis* and *Myotis blythii* at the colonies of Naters and Raron in relation to cockchafer years. The models for probabilities of recapture (*p*_Col*year+rep+spec_), movement between colonies (ψ_juv:.; ad: spec_) and age‐specific first time reproduction (α_a3+spec_) were always the same therefore not included in the model notation below. We present the model's deviance, the number of estimated parameters, the difference in the Akaike's information criterion between the actual and the best model (ΔAIC), and the Akaike's weight

Model for first‐year survival	Model for adult survival	Deviance	Parameters	ΔAIC	Weight
Year	*blythii*.:.; *myotis*: cockchafer	8747.12	52	0.00	0.261
Year	*blythii*.:.; *myotis*: .	8749.19	51	0.07	0.251
Year	*blythii*.: cockchafer; *myotis*:.	8748.47	52	1.35	0.132
Year	*blythii*.: cockchafer; *myotis*: cockchafer	8746.72	53	1.60	0.117
*blythii*.: cockchafer; *myotis*: year	*blythii*.:.; *myotis*: cockchafer	8746.43	54	3.31	0.049
*blythii*.: year; *myotis*: cockchafer	*blythii*.:.; *myotis*: cockchafer	8746.73	54	3.62	0.043
*blythii*.: cockchafer; *myotis*: year	*blythii*.:.; *myotis*:.	8749.20	53	4.08	0.034
*blythii*.: cockchafer; *myotis*: cockchafer	*blythii*.:.; *myotis*: cockchafer	8765.89	45	4.78	0.024
*blythii*.: cockchafer; *myotis*: year	*blythii*.: cockchafer; *myotis*: cockchafer	8746.16	55	5.05	0.021
*blythii*.: year; *myotis*: cockchafer	*blythii*.: cockchafer; *myotis*: cockchafer	8746.31	55	5.19	0.019
*blythii*.: cockchafer; *myotis*: year	*blythii*.: cockchafer; *myotis*:.	8748.67	54	5.55	0.016
*blythii*.: year; *myotis*: cockchafer	*blythii*.:.; *myotis*: .	8751.80	53	6.69	0.009
*blythii*.: cockchafer; *myotis*: cockchafer	*blythii*.: cockchafer; *myotis*: cockchafer	8765.87	46	6.75	0.009
*blythii*.: cockchafer; *myotis*: cockchafer	*blythii*.:.; *myotis*: .	8770.56	44	7.45	0.006
*blythii*.: year; *myotis*: cockchafer	*blythii*.: cockchafer; *myotis*: .	8750.99	54	7.87	0.005
*blythii*.: cockchafer; *myotis*: cockchafer	*blythii*.: cockchafer; *myotis*: .	8770.41	45	9.29	0.003

Model notation: year: different parameter for each year; cockchafer: different parameter for years with and without mass occurrence of cockchafers; *: interactive effects; + additive effects; . is for constancy.

Age‐specific probability to reproduce for the first time was not affected by mass cockchafer years at all (Table 4). Thus, individuals of both species did not have a higher probability to reproduce for the first time in a cockchafer year compared to a non‐cockchafer year. Moreover, whether or not an individual was born in a cockchafer year did not affect its probability to engage into first reproduction (cohort effects, i.e., anticipated cockchafer‐mediated first breeding).

**Table 4:** Modeling of age‐specific probability to start to reproduce in *Myotis myotis* and *Myotis blythii* at the colonies of Naters and Raron in relation to cockchafer years. The models for probabilities of recapture (*p*_Col*year+rep+spec_), movement between colonies (ψ_juv:.; ad: spec_) and survival (ϕ_juv: year; ad: spec_) were always the same and therefore not included in the model notation below. We present the model's deviance, the number of estimated parameters, the difference in the Akaike's information criterion between the actual and the best model (ΔAIC), and the Akaike's weight

Model for age‐specific probability to start reproduction (α)	Deviance	Parameters	ΔAIC	Weight
a3 + spec	8749.19	51	0.00	0.771
*blythii*.: a3; *myotis*: a3 + cockchafer(coh)	8747.92	54	4.73	0.072
*blythii*.: a3; *myotis*: a3 + cockchafer(time)	8748.98	54	5.79	0.043
*blythii*.: a3 + cockchafer(time); *myotis*: a3	8748.99	54	5.80	0.042
*blythii*.: a3 + cockchafer(coh); *myotis*: a3	8749.00	54	5.81	0.042
*blythii*.: a3; *myotis*: a3*cockchafer(coh)	8747.83	56	8.65	0.010
*blythii*.: a3; *myotis*: a3*cockchafer(time)	8748.45	56	9.26	0.008
*blythii*.: a3*cockchafer(coh); *myotis*: a3	8748.92	56	9.73	0.006
*blythii*.: a3*cockchafer(time); *myotis*: a3	8748.97	56	9.78	0.006

Model notation: year: different parameter for each year, a2: 2 age classes (1st year, later), a3: 3 age classes (1st year, 2nd year, later), cockchafer(time): different parameter for years with and without mass occurrence of cockchafers; cockchafer(coh): different parameter depending on whether the individual was born in a cockchafer or a non‐cockchafer year (cohort effect), * interactive effects, + additive effects,. is for constancy.

## 4 Discussion

This comparative demographic analysis of two closely related sympatric bat species corroborates several predictions derived from life‐history theory about the effects of interspecific niche differentiation on vital rates (Stearns, 1992). It also provides support to our hypotheses concerning the subtle vital rate adjustments that may be driven by temporal fluctuations in food availability (Pelisson, Bel‐Venner, Giron, Menu, & Venner, 2013).

First‐year survival probabilities fluctuated a lot over the years, but remained in concert and showed comparable magnitude for both species. This is in line with the observation that life‐history components with low fitness impact (low demographic sensitivity) vary more over time (Bjorkvoll et al., 2012; Gaillard & Yoccoz, 2003; Oli & Dobson, 2003; Pfister, 1998; Saether & Bakke, 2000; Schorcht et al., 2009). This large variability in first‐year survival is likely due to temporal variability in food supply mediated by weather conditions, which is known to impact the demographic trajectories of bat populations (Arlettaz et al., 2001; Frick et al., 2010; O'Shea, Ellison, & Stanley, 2011).

As reported for mammals in general (Descamps, Boutin, Berteaux, & Gaillard, 2006; Promislow & Harvey, 1990) and greater horseshoe bats in particular (Ransome, 1995; Schaub, Gimenez, Sierro, & Arlettaz, 2007), life‐history trade‐offs against age at first reproduction were probably the reason for higher adult survival probabilities in lesser than in greater mouse‐eared bats. Mean life expectation at 1 year of age is 5.6 years for lesser and 4.3 years for greater mouse‐eared bats (calculated as −1/ln(ϕ_ad_). An average lesser mouse‐eared bat can expect to have 3.6 years of reproductive opportunities during its life (accounting for the fact that its age at first reproduction is, on average, 3 years), whereas a greater mouse‐eared bat will have 3.3 reproductive years at its disposal (age at first reproduction: 2 years). As mouse‐eared bats attempt to breed every year, have a litter size of only one, and face identical preweaning mortality (Arlettaz, 1993; own unpublished data), lifetime reproductive success appears fairly identical (3.3 and 3.6) in the two species despite contrasted life‐history strategies. To the best of our knowledge, this would be the first indication ever that subtle life‐history modulations induced by different realized ecological niches provide similar lifetime breeding performances between closely related species.

The fact that adults move more between colonies than first‐year individuals may result from breeding as closely as possible to crucial foraging grounds—notably where there is sporadic mass prey supply, that is, availability of the pulse resource represented by cockchafers. (Arlettaz, 1996, 1999)—in order to optimize their energy balance. Such a drive does not exist in young individuals. Some adult females would thus be likely to select the nursery roost that is better located in the foraging landscape, even if mouse‐eared bats can exploit foraging grounds situated up to 25 km from their maternity roost (Arlettaz, 1999). The spatial patterns of movements between the two colonial roosts observed in this study were indeed asymmetrical, which can be further explained by spatial variation in prey supply and related energetic constraints linked to commuting flights. Adult lesser mouse‐eared bats preferentially moved from Raron to Naters, while the opposite was observed for adult greater mouse‐eared bats. The Raron colony harbored a greater proportion of greater mouse‐eared bats (79% of marked mouse‐eared bats in our dataset), while the Naters colony harbored proportionally more lesser mouse‐eared bats (57%). This is likely due to more bush cricket‐rich habitats around Naters and more carabid‐beetle habitats around Raron (Arlettaz, 1999), these two beetle taxa constituting the bulk of the prey for greater and lesser mouse‐eared bats, respectively (Arlettaz, Perrin, et al., 1997). The movements of adult females between the roosts would thus be driven mostly by the temporary availability of cockchafers locally, that is, in the surroundings of nursery roosts.

In contrast to the evidence that major niche differentiation affects survival and age at first reproduction while enabling a stable sympatric coexistence (Arlettaz, 1999; Arlettaz, Perrin, et al., 1997), the effects of site‐specific, year‐to‐year variation in cockchafers were less pronounced. Firstly, first‐year survival did not seem to be affected while adult survival did, although not in the expected direction with respect to species. In effect, based on both single model outcomes and model averaging, it seems that mass cockchafer availability is more likely to increase adult survival in greater mouse‐eared bats than in lesser mouse‐eared bats. Age at first reproduction was not positively influenced by cockchafer availability in a given year, nor were cohorts engaging into reproduction at an earlier age. Several aspects linked to the bat's divergent trophic ecology enable interpreting a posteriori from a functional viewpoint this observed pattern that contradicts our initial predictions. Greater mouse‐eared bats are predators of relatively large and hard chitinous coleopterans such as carabid beetles (Arlettaz & Perrin, 1995). They have stronger jaws and teeth than the lesser mouse‐eared bats (Ghazali & Dzeverin, 2013) that specialize on bush crickets with much softer exoskeletons (Arlettaz, Perrin, et al., 1997). Cockchafers have hard exoskeletons, as is typical for beetles. Although cockchafers are intensively exploited by lesser mouse‐eared bats during peak years—contrary to greater mouse‐eared bats which maintain a much broader diet— and constitute the bulk of their diet as long as bush crickets remain unavailable early in the season (Arlettaz, Perrin, et al., 1997; Arlettaz et al., 2001), they may not represent, as we initially thought, such an excellent alternative prey to bush crickets (Arlettaz et al., 2001). Cockchafers could even constitute a worse‐than‐nothing option for lesser mouse‐eared bats. A beetle specialist such as the greater mouse‐eared bat may thus benefit from the myriads of cockchafers, with positive effects mirroring in adult survival, whereas a slender predator specialized in softer arthropods may not. As greater mouse‐eared bats have access to their favorite food, carabid beetles, from their very first days of activity following hibernation (Arlettaz & Perrin, 1995), cockchafers would provide a slight advantage for this species, which is reflected in slightly enhanced vital rates. Furthermore, the reliance of *M. blythii* on cockchafers in some years makes them engage earlier into reproduction in cockchafer years as a result of an accelerated pregnancy (Arlettaz et al., 2001). This might cause problems if they then face low food availability during lactation, that is, in the period when cockchafers are no longer available while bush crickets—which have successive instars—have not yet reached the minimal critical body size for entering *M. blythii*'s diet (Arlettaz et al., 2001). Such situations of mismatch between patterns of resource availability and acquisition might be reflected in fitness costs, notably in adult survival probabilities as lactation represents the energetically most demanding period of a bat life cycle.

We conclude that major patterns in species‐specific prey availability and interaction with a clear‐cut interspecific trophic niche partitioning can result in diverging evolution of life‐history strategies. Beyond contrasted prey preferences, more subtle spatiotemporal fluctuations of prey availability had a less perceptible impact on life‐history components. As our two model species are true sibling species sharing a more recent common ancestor than any other pair of living bat species does, only niche partitioning can drive the major life‐history differences and subtle life‐history adjustments we observed at the interspecific level. Our results show that the classical life‐history trade‐offs typically observed within a species also operate at the interspecific level, showing that evolution of life‐history strategies is eventually governed by species‐specific patterns of trophic resource availability and acquisition during niche differentiation.

## Conflict of Interest

None declared.
