# Supplementary material for: Food availability as a major driver in the evolution of life‐history strategies of sibling species
Source: Ecol Evol. 2017 Apr 28;7(12):4163–72. doi: 10.1002/ece3.2909 (PMC5478057; doi:10.1002/ece3.2909)
Supplement: Supplementary file 1 [file ECE3-7-4163-s001.docx]

# Supplementary material: model selection stragegy

To test our various hypotheses about the variation in life history traits we formulated different models. Each model is specific to a hypothesis and some also represent alternative hypotheses. To make inferences from this set of models, we ranked them according to their support by the data by means of the Akaike’s information criterion (AIC) ([Burnham & Anderson 1998](#_ENREF_14)). To properly interpret the findings, we also computed the Akaike’s weight, which is the probability that a particular model is the best one, given the data and the set of candidate models ([Burnham & Anderson 1998](#_ENREF_14)).

We analysed the data of the two species simultaneously, such that the significance of species-specific differences could formally be assessed by step-by-step model selection. Starting from the most general models, we fitted several models for each parameter type (recapture, movement, age at first reproduction, survival) in turn, while keeping the other parameter types at a parsimonious structure. For each step we retained models within an ΔAIC of 2. Generally, we first focused on the variation of the parameters with respect to time, year, species and colony effects, testing whether the interspecific life history differences occurred as predicted. Second, we fitted additional models reflecting variations resulting from the mass cockchafer years in order to study how life history traits might be shaped by cyclic food conditions.

We applied the model notation as proposed by ([Lebreton *et al.* 1992](#_ENREF_26)). The symbol A_x*y_ denotes that the parameter A is modelled as a linear function of x and y, where x and y interact. The symbol A_x+y_ denotes that A is modelled as a linear function of x and y, but that there is no interaction between x and y.

We used software E-SURGE ([Choquet, Rouan & Pradel 2009](#_ENREF_17)) for modelling and parameter estimation and U-CARE ([Choquet *et al.* 2001](#_ENREF_16); [Choquet *et al.* 2009](#_ENREF_15)) for goodness-of-fit testing. The latter was performed for a general model where free movement between states was possible for all individuals. The standard errors of derived parameters were calculated with the delta method ([Seber 1982](#_ENREF_41)). To present the estimated parameters we conducted model averaging across all models having an Akaike weight of at least 0.02.

# Supplementary material: tables with model selection summaries

**Table S1.** Selection among different recapture probability models of *M. myotis* and *M. blythii* at the colonies of Naters and Raron. The models for probabilities of survival (φ_a2*species*year*colony_), movement between colonies (ψ_a2*species_) and age-specific first time reproduction (α_a3*species*colony_) were always the same and therefore not included in the model notation below. The subscripts refer to the factors in the models, where *rep* is a difference between individuals that have reproduced at least once and those that have not started to reproduce yet. We present the model’s deviance, the number of estimated parameters, the difference in the Akaike’s information criterion between the actual and the best model (ΔAIC), and the Akaike’s weight.

|  |  |  |  |  |
| --- | --- | --- | --- | --- |
| Recapture model (*p*) | Deviance | Parameters | ΔAIC | Weight |
|  |  |  |  |  |
|  |  |  |  |  |
| Colony*year+rep+species | 8643.33 | 137 | 0.00 | 0.470 |
| Colony*year*rep | 8606.60 | 156 | 1.27 | 0.249 |
| Colony*year+rep | 8647.23 | 136 | 1.91 | 0.181 |
| Colony*year*rep+species | 8604.42 | 158 | 3.10 | 0.100 |
| Colony*year*rep*species | 8543.48 | 199 | 24.15 | 0.000 |
|  |  |  |  |  |

**Table S2.** Selection among different models for movement probability of *M. myotis* and *M. blythii* between the colonies Naters and Raron. The models for probabilities of survival (φ_a2*species*year*colony_) and age-specific first time reproduction (α_a3*species*colony_) were always the same and therefore not included in the model notation below. For the recapture rate the models with ΔAIC < 2 from the previous step (Appendix A table 1) were used. We present the model’s deviance, the number of estimated parameters, the difference in the Akaike’s information criterion between the actual and the best model (ΔAIC), and the Akaike’s weight. See Appendix A, Table 1 for model notation.

|  |  |  |  |  |  |
| --- | --- | --- | --- | --- | --- |
| Movement model (ψ) | Recapture model (*p*) | Deviance | Parameters | ΔAIC | Weight |
|  |  |  |  |  |  |
|  |  |  |  |  |  |
| juv:.; ad: species | Colony*year+rep+species | 8645.65 | 135 | 0.00 | 0.361 |
| juv:.; ad: species | Colony*year*rep | 8608.87 | 154 | 1.22 | 0.196 |
| a2*species | Colony*year+rep+species | 8643.36 | 137 | 1.70 | 0.154 |
| juv:.; ad: species | Colony*year+rep | 8649.48 | 134 | 1.83 | 0.145 |
| a2*species | Colony*year*rep | 8606.60 | 156 | 2.95 | 0.083 |
| a2*species | Colony*year+rep | 8647.23 | 136 | 3.58 | 0.060 |
| a2 | Colony*year+rep+species | 8674.08 | 133 | 24.43 | 0.000 |
| a2 | Colony*year+rep | 8677.30 | 132 | 25.65 | 0.000 |
| juv: species; ad: . | Colony*year+rep+species | 8671.74 | 135 | 26.09 | 0.000 |
| a2 | Colony*year*rep | 8638.45 | 152 | 26.80 | 0.000 |
| juv: species; ad: . | Colony*year+rep | 8674.98 | 134 | 27.33 | 0.000 |
| juv: species; ad: . | Colony*year*rep | 8636.26 | 154 | 28.71 | 0.000 |
|  |  |  |  |  |  |

**Table S3.** Selection among different models for age-specific probability to start to reproduce of *M. myotis* and *M. blythii* at the colonies of Naters and Raron. The model for survival probability (φ_a2*species*year*colony_) was always the same and therefore not included in the model notation below. We present the model’s deviance, the number of estimated parameters, the difference in the Akaike’s information criterion between the actual and the best model (ΔAIC), and the Akaike’s weight. See appendix A table 1 for model notation.

|  |  |  |  |  |  |  |
| --- | --- | --- | --- | --- | --- | --- |
| First time breeding model (α) | Movement model (ψ) | Recapture model (*p*) | Deviance | Parameters | ΔAIC | Weight |
|  |  |  |  |  |  |  |
|  |  |  |  |  |  |  |
| a3+species | juv:.; ad: species | Colony*year+rep+species | 8646.74 | 127 | 0.00 | 0.358 |
| a3+species | juv:.; ad: species | Colony*year*rep | 8609.73 | 146 | 0.99 | 0.218 |
| a3+species | a2*species | Colony*year+rep+species | 8644.45 | 129 | 1.72 | 0.152 |
| a3+species | juv:.; ad: species | Colony*year+rep | 8650.57 | 126 | 1.84 | 0.143 |
| a3*species | juv:.; ad: species | Colony*year+rep+species | 8646.62 | 129 | 3.88 | 0.051 |
| a3*species | juv:.; ad: species | Colony*year*rep | 8909.50 | 148 | 4.77 | 0.033 |
| a3*species | a2*species | Colony*year+rep+species | 8644.28 | 131 | 5.54 | 0.022 |
| a3*species | juv:.; ad: species | Colony*year+rep | 8650.40 | 128 | 5.66 | 0.021 |
| a3*species*colony | juv:.; ad: species | Colony*year+rep+species | 8645.65 | 135 | 14.89 | 0.000 |
| a3*species*colony | juv:.; ad: species | Colony*year*rep | 8608.87 | 154 | 16.13 | 0.000 |
| a3*species*colony | a2*species | Colony*year+rep+species | 8643.36 | 137 | 16.59 | 0.000 |
| a3*species*colony | juv:.; ad: species | Colony*year+rep | 8649.48 | 134 | 16.75 | 0.000 |
| a3 | juv:.; ad: species | Colony*year*rep | 8650.62 | 145 | 39.88 | 0.000 |
| a3 | juv:.; ad: species | Colony*year+rep+species | 8689.71 | 126 | 40.99 | 0.000 |
| a3 | juv:.; ad: species | Colony*year+rep | 8693.18 | 125 | 42.45 | 0.000 |
| a3 | a2*species | Colony*year+rep+species | 8687.40 | 128 | 42.67 | 0.000 |
|  |  |  |  |  |  |  |

Table S4. Selection among different models for survival probability of *M. myotis* and *M. blythii* at the colonies of Naters and Raron. The model for age-specific first time reproduction (α_a3+species_) was always the same and therefore not included in the model notation below. We present the model’s deviance, the number of estimated parameters, the difference in the Akaike’s information criterion between the actual and the best model (ΔAIC), and the Akaike’s weight. Model notation: *rep:* individuals that have reproduced at least once and those that have not started to reproduce yet differ; *a2*: 2 age classes (1. year, later); *a3*: 3 age classes (1. year, 2. year, later); *year:* different parameter for each year; *Col:* different parameter for each colony; *spec:* different parameter for each species; *juv:* juveniles (1st year); *ad:* adults (at least 1 year old), *: interactive effects, +: additive effects, . is for constancy.

|  |  |  |  |  |  |  |
| --- | --- | --- | --- | --- | --- | --- |
| Survival model (φ) | Movement model (ψ) | Recapture model (*p*) | Deviance | Parameters | ΔAIC | Weight |
|  |  |  |  |  |  |  |
|  |  |  |  |  |  |  |
| juv: year; ad: spec | juv:.; ad: spec | Col*year+rep+spec | 8749.19 | 51 | 0.00 | 0.212 |
| juv: year; ad: spec | a2*spec | Col*year+rep+spec | 8745.79 | 53 | 0.60 | 0.157 |
| juv: year; ad: spec | juv:.; ad: spec | Col*year*rep | 8710.05 | 71 | 0.86 | 0.138 |
| juv: year+spec; ad: spec | juv:.; ad: spec | Col*year+rep+spec | 8749.12 | 52 | 1.93 | 0.081 |
| juv: year+spec; ad: spec | juv:.; ad: spec | Col*year*rep | 8709.25 | 72 | 2.06 | 0.076 |
| juv: year+spec; ad: spec | a2*spec | Col*year+rep+spec | 8745.61 | 54 | 2.42 | 0.063 |
| juv: year*spec; ad: spec | juv:.; ad: spec | Col*year*rep | 8690.11 | 82 | 2.92 | 0.049 |
| juv: year; ad: . | juv:.; ad: spec | Col*year+rep+spec | 8754.83 | 50 | 3.64 | 0.034 |
| juv: year; ad: . | a2*spec | Col*year+rep+spec | 8751.29 | 52 | 4.10 | 0.027 |
| a2*spec | a2*spec | Col*year+rep+spec | 8767.44 | 44 | 4.25 | 0.025 |
| a2*spec | juv:.; ad: spec | Col*year+rep+spec | 8771.52 | 42 | 4.33 | 0.024 |
| juv: year*spec; ad: spec | juv:.; ad: spec | Col*year+rep+spec | 8731.57 | 62 | 4.39 | 0.024 |
| juv: year+spec; ad: . | juv:.; ad: spec | Col*year+rep+spec | 8754.83 | 51 | 5.61 | 0.013 |
| juv: year+spec; ad: . | juv:.; ad: spec | Col*year*rep | 8714.99 | 71 | 5.80 | 0.012 |
| juv: year; ad: . | juv:.; ad: spec | Col*year*rep | 8717.05 | 70 | 5.86 | 0.011 |
| juv: year*spec; ad: spec | a2*spec | Col*year+rep+spec | 8729.07 | 64 | 5.88 | 0.011 |
| juv: year+spec; ad: . | a2*spec | Col*year+rep+spec | 8751.29 | 53 | 6.10 | 0.010 |
| juv: year; ad: spec | a2*spec | Col*year+rep | 8757.74 | 50 | 6.55 | 0.008 |
| a2+year | juv:.; ad: spec | Col*year+rep+spec | 8778.82 | 40 | 7.63 | 0.005 |
| a2+year | a2*spec | Col*year+rep+spec | 8774.99 | 42 | 7.80 | 0.004 |
| juv: year+spec; ad: spec | a2*spec | Col*year+rep | 8757.66 | 51 | 8.47 | 0.003 |
| juv: year*spec; ad: . | juv:.; ad: spec | Col*year*rep | 8698.20 | 81 | 9.01 | 0.002 |
| juv: year*spec; ad: . | juv:.; ad: spec | Col*year+rep+spec | 8738.54 | 61 | 9.35 | 0.002 |
| juv: year*spec; ad: spec | a2*spec | Col*year+rep | 8739.72 | 61 | 10.53 | 0.001 |
| juv: year*spec; ad: . | a2*spec | Col*year+rep+spec | 8735.88 | 63 | 10.69 | 0.001 |
| a2*spec+year | a2*spec | Col*year+rep+spec | 8752.33 | 55 | 11.14 | 0.001 |
| a2+spec+year | juv:.; ad: spec | Col*year+rep+spec | 8759.28 | 52 | 12.09 | 0.001 |
| a2+spec+year | a2*spec | Col*year+rep+spec | 8755.37 | 54 | 12.18 | 0.000 |
| a2*spec+year | juv:.; ad: spec | Col*year+rep+spec | 8756.55 | 53 | 12.36 | 0.000 |
| a2*spec | a2*spec | Col*year+rep | 8781.78 | 41 | 12.59 | 0.000 |
| a2 | a2*spec | Col*year+rep+spec | 8757.87 | 53 | 12.70 | 0.000 |
| juv: year; ad: . | a2*spec | Col*year+rep | 8766.14 | 49 | 12.95 | 0.000 |
| a2 | juv:.; ad: spec | Col*year+rep+spec | 8762.51 | 51 | 13.32 | 0.000 |
| juv: year+spec; ad: . | a2*spec | Col*year+rep | 8765.35 | 50 | 14.17 | 0.000 |
| a2*year | juv:.; ad: spec | Col*year*rep | 8708.73 | 79 | 15.54 | 0.000 |
| a2*year | juv:.; ad: spec | Col*year+rep+spec | 8748.26 | 60 | 17.07 | 0.000 |
| a2*spec | juv:.; ad: spec | Col*year*rep | 8744.48 | 62 | 17.30 | 0.000 |
| a2+year | a2*spec | Col*year+rep | 8790.70 | 39 | 17.51 | 0.000 |
| a2*year | a2*spec | Col*year+rep+spec | 8744.80 | 62 | 17.61 | 0.000 |
| a2+spec+year | a2*spec | Col*year+rep | 8767.09 | 51 | 17.90 | 0.000 |
| juv: year*spec; ad: . | a2*spec | Col*year+rep | 8749.27 | 60 | 18.08 | 0.000 |
| a2*spec+year | a2*spec | Col*year+rep | 8765.31 | 52 | 18.13 | 0.000 |
| a2+spec+year | juv:.; ad: spec | Col*year*rep | 8727.15 | 72 | 19.97 | 0.000 |
| a2+year | juv:.; ad: spec | Col*year*rep | 8751.41 | 60 | 20.22 | 0.000 |
| a2*spec+year | juv:.; ad: spec | Col*year*rep | 8726.26 | 73 | 21.07 | 0.000 |
| a2 | a2*spec | Col*year+rep | 8773.84 | 50 | 22.65 | 0.000 |
| a2*spec*year | juv:.; ad: spec | Col*year*rep | 8772.24 | 101 | 23.05 | 0.000 |
| a2 | juv:.; ad: spec | Col*year*rep | 8733.14 | 71 | 23.95 | 0.000 |
| a2*year | a2*spec | Col*year+rep | 8760.04 | 59 | 26.85 | 0.000 |
| a2*spec*year | juv:.; ad: spec | Col*year+rep+spec | 8716.06 | 82 | 28.87 | 0.000 |
| a2*spec*year | a2*spec | Col*year+rep+spec | 8713.44 | 84 | 30.25 | 0.000 |
| a2*spec*year | a2*spec | Col*year+rep | 8721.51 | 81 | 32.32 | 0.000 |
| a2*spec*year*col | juv:.; ad: spec | Col*year+rep+spec | 8646.81 | 127 | 49.62 | 0.000 |
| a2*spec*year*col | juv:.; ad: spec | Col*year*rep | 8609.73 | 146 | 50.54 | 0.000 |
| a2*spec*year*col | a2*spec | Col*year+rep+spec | 8644.45 | 129 | 51.35 | 0.000 |
| a2*spec*year*col | a2*spec | Col*year+rep | 8650.61 | 126 | 51.42 | 0.000 |
